# Is there a sexual difference in the relationship between sociodemographic information and the unmet dental care needs of disabled adults? An analysis from a national survey on persons with disabilities

**DOI:** 10.1186/s12903-023-03576-w

**Published:** 2023-11-03

**Authors:** Na-Hyeon Kim, Jae-In Ryu

**Affiliations:** 1 Department of Dentistry, Kyung Hee University College of Dentistry, Seoul, Republic of Korea; 2 Department of Preventive and Social Dentistry, Kyung Hee University College of Dentistry, Seoul, Republic of Korea

**Keywords:** Disabled person, Dental care for disabled, Dental health services, Health services for persons with disabilities, Social security

## Abstract

**Background:**

Health status and health care utilization in people with disabilities are more likely to be poorer than those without disabilities. Previous studies showed that there were gaps in health-related conditions by sociodemographic information and gender but the association between these factors was not explained. This study aims to analyze the relationship between sociodemographic information and the unmet dental care needs of people with disabilities and explore the effect of sex within this relationship.

**Methods:**

The 2014 national survey on persons with disabilities was used, which separated unmet healthcare needs into medical and dental services. Unweighted samples included 6,824 people with disabilities in total and 6,555 (96.1% of the total, weighted as 6,583) people aged 20 years or older were selected as the study population. Frequency and chi-square tests were conducted to determine differences in the prevalence of unmet dental needs based on socioeconomic information, chronic diseases, and behavioral factors after applying weights. Logistic regressions were performed using an adjusted model with sociodemographic information, chronic diseases, and behavioral variables. All analyses were performed using IBM SPSS Statistics for Windows, version 26 (IBM Corp., Armonk, N.Y., USA).

**Results:**

Analysis of the sociodemographic factors related to unmet dental care revealed that they were higher in women and the elderly. In the fully adjusted logistic model, most of the sociodemographic information was significantly associated with unmet dental needs. The lowest group was 4.18 times more likely to have unmet dental care needs than the richest group, and females and middle-school graduates were almost twice as likely to experience unmet dental care needs than males and university graduates. Considering the interaction effect of age on unmet dental needs depending on sex differences, the odds ratio decreased for females with every annual increase in both models. Compared with the younger group, the older group showed a lower risk of having unmet dental needs, especially in females.

**Conclusions:**

The factor most closely related to the unmet dental care needs of disabled people was socioeconomic problems. Its influence also differed by sex and age. Therefore, economic support measures and sexual differences are needed for long-term policy consideration to reduce the unmet dental care needs of disabled people.

**Supplementary Information:**

The online version contains supplementary material available at 10.1186/s12903-023-03576-w.

## Background

Approximately 1.3 billion people have disabilities, 16% of the world’s population in 2021, which has increased by 300 million in the last decade [1]. The prevalence of disabilities increases with age, such that 34.4% were those over sixty years. Moreover, women showed a slightly higher proportion than men (18.0% versus 14.2%, respectively). Disabilities have diverse impacts on people not only their health, but also for example low educational opportunities, low income, and high unemployment [2, 3]. This resulted in health inequality through direct and indirect effects [4]. People with disabilities are more likely to be poorer in health status, health-risk behaviors, preventive healthcare utilization, and chronic health conditions than those without disabilities [5].

Oral diseases in disabled people rapidly progress to serious conditions [1, 2, 6–9], such as a higher Decayed, Missing, Filled Teeth (DMFT) index or tooth loss [10]. Oral health affects basic functions, such as chewing, swallowing, pronunciation, and breathing [11, 12], and the quality of life through its close relationship with systemic health and psychosocial interactions [13, 14]. The oral health status of the world’s population has improved [15]; however, inequality still exists [16–19]. The group with lower socioeconomic status (SES) showed higher levels of prevalence in oral disease, such as untreated dental caries, periodontal disease, or oral cancer. Although this might be common in most countries, it must be considered an “unfair, unjust, and unacceptable” situation; thus, appropriate measures must be taken to improve this [20].

Vulnerable people are often exposed to poor environments, have poor self-care skills, or face difficulties in accessing dental care [21, 22]. The higher barriers in physical or traveling, communication, and financial support [23, 24] or low prioritization of dental services [25], the worse access to healthcare. As a result, they are less likely to receive preventive treatments, such as oral examinations [26], and more likely to need dental care, such as prosthetic treatment [27].

Unmet need is defined as “difficulties receiving service in response to a problem that significantly interferes with daily life” [28, 29]. Disability was suggested as the most important factor of unmet need for medical care [30]. Moreover, disabilities interact with healthcare access, which is influenced by personal and environmental factors [31]. A study from Greece showed that unmet needs for dental care were 1.8 times higher in disabled people than those who were not, and approximately 25% of disabled people did not receive dental care for economic reasons [32]. Socioeconomic factors, such as income level, were identified as one of the important reasons for this gap [33–35]. Furthermore, there was a gender gap within the disabled group, women had a higher rate of unmet need for medical care than men [36]. Women with disabilities showed a 7.2 times higher rate of unmet needs for healthcare services than men without disabilities. However, there are lack of studies that consider various sociodemographic information and sex for the unmet dental care needs of persons with disabilities using a national samples survey.

This study aims to analyze the relationship between sociodemographic information and unmet dental care needs and to investigate its differences among male and female adults with disabilities in Korea.

## Methods

### Study design and participants

The national survey on persons with disabilities began in 1985 and has been conducted every three years since 2005. It identifies living conditions and welfare needs to develop a national policy and implementational plan. In this study, the 2014 survey data were used [37], which separated unmet healthcare needs into medical and dental services. The survey was conducted between August and October 2014. The sampling frame was 90% of the 2010 Census on Population and Housing units, and this study extracted the sampling units using the proportional stratification probability method. The number of people in each sampling unit was determined by proportional distribution according to the square roots of the registered disabled people numbers in 16 cities or provinces. The sampling household was confirmed as 48,344 within 1,004 extracted units and 251 preparatory units for cases of non-response, rejection, and redevelopment. Finally, 38,560 households completed the survey, with a response rate of 79.8%. The surveyed participants were 104,703, including disabled and non-disabled together. Among them, unweighted samples with disabilities were 6,824 people, and 6,555 (96.1% of the total, weighted as 6,583) people aged 20 years or older were selected as the study population. The final weight was calculated by multiplying the design, non-response adjustment, and post-stratification weights. The final weight for the disabled was calculated by post-adjusting the weight for the disabled by age, sex, type of disability, and 16 regions, using information for the unregistered disabled in December 2013.

The survey was conducted after obtaining the participants’ informed consent. There was a type of disability that had difficulty communicating during the survey. In this case, the responses were received through family members (mainly spouses and parents) in households. As a result, 78.5% of all disabled people responded by themselves and 21.5% were proxy responses from their family members. The study was approved by the Research Ethics Review Committee of the Korea Institute of Health and Social Affairs (KIHASA) (IRB No. KIHASA IRB-2014-14). This analytical study was approved again by the institutional review board of Kyung Hee University (IRB No. KHSIRB-22-442) and given exemption from review because the retrospective analysis included the dataset from a national survey and did not contain personally identifiable information. All methods were performed following the survey guidelines and regulations.

### Statistical analysis

The questions have developed since the start of the national survey on persons with disabilities. They were supplemented and revised by the government and the professionals in this field. The outcome variable was the unmet dental care need with responses such as, “I thought I needed dental treatments for the past year, but I have not received them.” This item adapted from the Korea National Health and Nutrition Examination Survey, which was proved validity and reliability. The independent variables were related to the unmet dental care needs and categorized into sociodemographic and health-related behavioral factors: (1) sociodemographic factors, such as sex, age, subjective economic class, health insurance type, and academic background; and (2) chronic disease and behavioral factors, such as hypertension, diabetes, smoking, frequency of toothbrushing per day, and oral examination. The patients were divided into three age groups: 20–39, 40–64, and 65 years. The persons with disabilities were defined, and the grades and types of disabilities were classified according to the Act on Welfare of Persons with Disabilities at the time of the investigation. Disability severity was categorized into two groups, 1st to 3rd (severe) and 4th to 6th (not severe) grade. Disabilities were classified as physical (physical, brain lesions; visual, hearing impairment; speech, kidney, heart, respiratory, liver, facial, intestinal, urinary, or epilepsy disorders) or mental (intellectual, autistic, or mental disorders). All the grades and standards of persons with disabilities were classified according to the Act of Welfare of Persons with Disabilities Act (see appendix). Health coverage was grouped into two, National Health Insurance (NHI) and Medical Aid.

Frequency and chi-square tests were conducted to determine differences in the prevalence of unmet dental needs based on socioeconomic information, chronic diseases, and behavioral factors after applying weights. Logistic regressions were performed using an adjusted model with sociodemographic variables only (Model 1) and a fully adjusted model with sociodemographic information, chronic diseases, and behavioral variables (Model 2). Variables that were not significant in the chi-square test were excluded from the logistic regression analysis. In the case of age, changes in the unmet dental care needs were not suitable when grouped; therefore, actual measurements of every age group were used in the logistic regression analysis. All figures in the table are weighted values. To determine the correlation between the variables in the model, the Variance Inflation Factor (VIF) was analyzed. All values were less than 10; therefore, multicollinearity was not observed. All analyses were performed using IBM Statistical Packages for Social Science (SPSS) Statistics for Windows, version 26 (IBM Corp., Armonk, N.Y., USA). Statistical significance was set to α = 0.05.

## Results

Among the survey population of persons with disabilities aged 20 years or older, more than half were men (57.5%) (Table 1). The middle-aged (40 to 64 years) and elderly (65 years and older) groups were slightly similar and the most common in the distribution (44.9% and 44.7%, respectively). Most of the disabilities were mild (62.8%) or physical (90.5%). Almost two-thirds of the participants had the lowest subjective SES, compared with the upper class (at least 0.9%). In terms of academic background, only 13.0% of the disabled participants graduated from university and almost four times had just finished their education in middle school (59.1%). Chronic diseases, such as hypertension or diabetes, and unhealthy behaviors, such as smoking, tooth brushing less than twice, or having no experience of oral examination in the last year, were minor in this study. Overall, one-quarter (27.5%) of the study population experienced unmet dental care needs. The distributional differences by sex in sociodemographic information were statistically significant in terms of age, educational level, health coverage, and most health-related behavioral factors except for the experience of oral examinations last year, and unmet dental care needs (p < 0.05).

**Table 1:** The weighted prevalence of study population of adult disabled

	Total		Males		Females		P value
	N	(%)	n	(%)	n	(%)	
Total	6,583	(100.0)	3,784	(57.5)	2,799	(42.5)	
Sociodemographic information							
Age							
20–44	1,072	(16.3)	719	(19.0)	353	(12.6)	< 0.001
45–64	2,569	(39.0)	1,706	(45.1)	863	(30.8)	
≥ 65	2,944	(44.7)	1,360	(35.9)	1,584	(56.6)	
Grade of disability							
4 ~ 6th	3,970	(62.8)	2,283	(62.4)	1,687	(63.3)	0.444
1 ~ 3rd	2,356	(37.2)	1,378	(37.6)	978	(36.7)	
Type of disability							
Mental	603	(9.5)	342	(9.3)	261	(9.8)	0.543
Physical	5,724	(90.5)	3,320	(90.7)	2,404	(90.2)	
Educational level							
University	854	(13.0)	660	(17.4)	195	(7.0)	< 0.001
High school	1,835	(27.9)	1,301	(34.4)	534	(19.1)	
Middle school	3,892	(59.1)	1,824	(48.2)	2,068	(73.9)	
Subjective SES							
Upper	56	(0.9)	35	(0.9)	21	(0.8)	0.737
Middle	2,071	(31.5)	1,187	(31.4)	885	(31.6)	
Low	4,457	(67.7)	2,563	(67.7)	1,894	(67.6)	
Health coverage							
NHI	5,540	(84.3)	3,197	(84.6)	2,343	(83.8)	0.005
Medicaid	1,033	(15.7)	580	(15.4)	453	(16.2)	
Chronic diseases or behaviors							
Hypertension							
No	3,871	(58.8)	2,361	(62.4)	1,510	(53.9)	< 0.001
Yes	2,713	(41.2)	1,424	(37.6)	1,289	(46.1)	
Diabetes							
No	5,290	(80.3)	3,072	(81.2)	2,217	(79.2)	0.046
Yes	1,294	(19.7)	712	(18.8)	582	(20.8)	
Smoking							
No	5,229	(79.4)	2,539	(67.1)	2,690	(96.1)	< 0.001
Yes	1,354	(20.6)	1,245	(32.9)	109	(3.9)	
Frequency of toothbrushing							
≥ 2	5,460	(82.9)	3,100	(81.9)	2,360	(84.3)	0.010
< 2	1,124	(17.1)	685	(18.1)	439	(15.7)	
Oral exam last year							
Yes	2,181	(33.1)	1,288	(34.0)	893	(31.9)	0.070
No	4,403	(66.9)	2,497	(66.0)	1,906	(68.1)	
Dental unmet need							
No	4,773	(72.5)	2,785	(73.6)	1,988	(71.0)	0.021
Yes	1,811	(27.5)	999	(26.4)	811	(29.0)	

Analysis of the factors related to unmet dental care revealed that they were higher in women and the elderly (Table 2). Statistically significant differences were not observed in the severity and type of disability between the two groups. Those with lower economic class, educational level, or medical coverage for the poor such as Medicaid had higher proportions of unmet dental care experience. In addition, a higher incidence of unmet dental care needs was observed in participants with chronic diseases or unhealthy behaviors.

**Table 2:** The weighted prevalence of unmet dental needs according to characteristics of the study population of adult disabled

	Total			P value	Male			P value	Female			P value
	N	n	(%)		N	n	(%)		N	n	(%)	
Total	6,583	1,810	(27.5)	0.021	3,784	999	(26.4)	0.021	2,799	811	(29.0)	
Sociodemographic information												
Age												
20–44	1,071	161	(15.0)	< 0.001	719	109	(15.2)	< 0.001	352	52	(14.8)	< 0.001
45–64	2,569	746	(29.0)		1,706	462	(27.1)		863	284	(32.9)	
≥ 65	2,944	904	(30.7)		1,359	428	(31.5)		1,584	475	(30.0)	
Grade of disability												
4 ~ 6th	3,970	1,055	(26.6)	0.102	2,283	577	(25.3)	0.103	1,687	478	(28.3)	0.540
1 ~ 3^rd^												
Type of disability	2,357	671	(28.5)		1,378	382	(27.7)		978	288	(29.4)	
Mental	5,724	1,577	(27.6)	0.136	3,320	884	(26.6)	0.064	2,404	693	(28.8)	0.843
Physical	603	149	(24.7)		341	75	(22.0)		262	74	(28.2)	
Educational level												
University	854	113	(13.2)	< 0.001	660	85	(12.9)	< 0.001	194	28	(14.4)	< 0.001
High school	1,835	466	(25.4)		1,301	307	(23.6)		534	159	(29.8)	
Middle school	3,892	1,230	(31.6)		1,824	608	(33.3)		2,068	623	(30.1)	
Subjective SES												
Upper	56	5	(8.9)	< 0.001	35	5	(14.3)	< 0.001	21	0	(0.0)	< 0.001
Middle	2,071	314	(15.2)		1,187	172	(14.5)		885	143	(16.2)	
Low	4,457	1,492	(33.5)		2,563	823	(32.1)		1,894	669	(35.3)	
Health coverage												
NHI	5,540	1,423	(25.7)	< 0.001	3,197	779	(24.4)	< 0.001	2,343	644	(27.5)	< 0.001
Medicaid	1,033	383	(37.1)		580	218	(37.6)		452	165	(36.5)	
Chronic diseases or behaviors												
Hypertension												
No	3,871	1,002	(25.9)	< 0.001	2,361	568	(24.1)	< 0.001	1,510	434	(28.7)	0.735
Yes	2,713	809	(29.8)		1,423	431	(30.3)		1,289	378	(29.3)	
Diabetes												
No	5,290	1,368	(25.9)	< 0.001	3,072	764	(24.9)	< 0.001	2,218	604	(27.2)	< 0.001
Yes	1,294	443	(34.2)		713	236	(33.1)		582	208	(35.7)	
Smoking												
No	5,230	1,399	(26.7)	0.007	2,539	635	(25.0)	0.006	2,690	763	(28.4)	< 0.001
Yes	1,354	412	(30.4)		1,245	364	(29.2)		109	48	(44.0)	
Frequency of toothbrushing												
≥ 2	5,461	1,401	(25.7)	< 0.001	3,100	757	(24.4)	< 0.001	2,361	644	(27.3)	< 0.001
< 2	1,123	410	(36.5)		685	243	(35.5)		439	168	(38.3)	
Oral exam last year												
Yes	2,181	458	(21.0)	< 0.001	1,288	271	(21.0)	< 0.001	893	187	(20.9)	< 0.001
No	4,403	1,353	(30.7)		2,497	729	(29.2)		1,906	624	(32.7)	

Logistic regression analysis for the factors that affect the prevalence of unmet dental care needs among adults with disabilities was presented in Table 3. All sociodemographic information including sex, age, subjective SES, educational level, and health coverage were significantly associated with unmet dental needs in Model 1. Only the middle-class group did not show a significant difference from the richest group. However, the lowest group was 4.5 times more likely to have unmet dental care needs than the richest group, and females and middle-school graduates were almost twice as likely to experience unmet dental care needs than males and university graduates. After adjusting for sociodemographic information and chronic diseases or behaviors together in Model 2, disabled adults showed similar results as Model 1. They show statistically significant differences in all included factors except the people who were in the middle SES (versus higher) or diagnosed (versus not diagnosed) with hypertension. Subjective SES and educational level showed similar odds ratios in this model. People with diabetes and unhealthy behaviors such as smoking, less frequency of toothbrushing, or no experience of oral exams had significant differences but below 1.5 odds ratio. Considering the interaction effect of age on unmet dental needs depending on sex differences, the odds ratio decreased for females with every annual increase in both models. Compared with the younger group, the older group showed a lower risk of having unmet dental needs (0.99 times having them a year), especially in females. The coefficient of determination of Nagel-Kerke’s R2 was 0.085 in Model 1 and 0.104 in Model 2, which was an increase of 0.019 from the previous model. The p-value was less than 0.01 for the test of model effects of interaction in both models between differences in sex and age.

**Table 3:** Odds ratio (OR) and 95% confidence interval (CI) estimated from logistic regression model for unmet dental needs of adults disabled

		Model 1			Model 2		
		OR	(95% CI)	P value	OR	(95% CI)	P value
Sociodemographic information							
Sex (male)							
female		2.04	(1.24–3.36)	0.005	2.62	(1.57–4.37)	< 0.001
Age							
1 year		1.02	(1.01–1.02)	< 0.001	1.02	(1.01–1.02)	< 0.001
Educational level (university)							
high school		1.85	(1.47–2.32)	< 0.001	1.80	(1.43–2.26)	< 0.001
middle school		2.05	(1.64–2.57)	< 0.001	1.93	(1.54–2.42)	< 0.001
Subjective SES (upper)							
Middle		1.80	(0.70-0.64)	0.222	1.74	(0.68–4.50)	0.251
Low		4.51	(1.76–11.56)	0.002	4.18	(1.63–10.74)	0.003
Health coverage (NHI)							
Medicaid		1.35	(1.17–1.56)	< 0.001	1.26	(1.08–1.46)	0.003
Chronic diseases or behaviors							
Hypertension (no)							
Yes					0.99	(0.87–1.12)	0.867
Diabetes (no)							
Yes					1.35	(1.17–1.55)	< 0.001
Smoking (no)							
Yes					1.32	(1.14–1.54)	< 0.001
Frequency of toothbrushing (≥ 2)							
< 2					1.34	(1.16–1.55)	< 0.001
Oral exam last year (yes)							
No					1.48	(1.30–1.68)	< 0.001
Interaction effects							
Sex*Age							
female*1 year		0.99	(0.98-1.00)	0.003	0.99	(0.98–0.99)	< 0.001
NagelKerke *R*^*2*^		0.085			0.104		

## Discussion

Factors related to unmet dental care experience among disabled adults were identified; men and women had large gaps in socioeconomic factors.

Subjective SES was the most significantly related factor in the unmet dental care needs of people with disabilities in Korea. 79.7% of the respondents answered that the reason for their unmet dental care needs was economic difficulty. In the case of medical care, half (58.8%) responded similarly [37]. As a representative population of the Korean National Health and Nutrition Examination Survey (KNHANES) only 11.0% answered similarly [38]. Disabled people experienced a seven-fold higher proportion than the national sample for the reason of unmet needs as financial difficulties as previous study [39]. Economic factors had the biggest impact on unmet dental care needs [40–42] even for people with disabilities, a lower household income indicated a higher need for treatment but a lower utilization rate of dental services [43–47]. The main causes of unmet medical care were poor income, educational background, or insurance [48, 49]. To improve access to dental treatment, the barriers due to financial or systematic difficulties for vulnerable people must be eliminated priorly [50–52]. The extended coverage of national health insurance could be another consideration to ease the financial burden [53, 54].

Second, differences in sex were observed; women were 2.6 times more likely to have unmet dental care needs than men. This can be interpreted as such that women had difficulties accessing dental care in general. However, the probability of experiencing unmet dental care needs in women inversely increased at the interaction terms with age; 0.99 times increments in odds ratios as the 1-year-old unit increased. The proportion with women had 2.6 times higher probability than men but also tended to decrease with age of having unmet dental needs after controlling for all factors including interaction terms in the final model. This might come from the descriptive discrepancy, the proportion of men with unmet dental care needs continued to increase with age, but that of women was not similar, as the highest were middle-aged (45–64 years) and decreased after 65 years. The general difficulties of women with disabilities originate from socioeconomic vulnerabilities [55]. The educational gap between middle school (73.9%) and university (7.0%) graduates was 10.6-fold in this study, which was five times higher than that for men (43.2% and 17.4%, 2.5-fold, respectively). Women, especially those with disabilities, are more likely to be vulnerable due to a lack of educational opportunities, which makes them more likely to be exposed to financial difficulties. However, ironically, there was no statistically significant difference in subjective SES between men and women. As income tends to be evaluated per household, it can be an indicator of households rather than individuals. The low educational status of individual women with disabilities might be influential to the possibility of unmet dental care needs due to physical, economic, and social disadvantages. Unmet dental care needs must be reduced through the provision of financial support for the disabled, prior to women in the short term, and the provision of extended opportunities for education and decent jobs in the long term.

Finally, those who did not visit a dental clinic for oral examination within a year were more likely to experience unmet dental care needs. Disabled people generally showed a lower chance of having oral examinations than non-disabled people; for example, only two-thirds of that in non-disabled people [56]. Most people who did not have oral exams answered the reason as they had no symptoms or thought they were healthy, and one-fifth of them said they had moving difficulty. When they have difficulties in using dental care services including oral examinations, unmet dental care needs are likely to increase. According to a recent national survey, the number of registered persons with disabilities in Korea continues to increase, and the proportion of elderly people aged 65 years is steadily increasing [57]. Thus, the government must be made aware of the importance of oral examinations which could be the beginning of the utilization of dental care, especially for people with disabilities.

The limitations of this study are as follows. First, it was unable to compare oral health status with unmet dental care needs because this survey was questionnaire-based. Second, this study used data from the survey on persons with disabilities. The analysis has limitations on the relative comparison of the study population with the whole population for unmet dental care needs. Further studies are necessary to analyze the data including oral health status using, for example, the National Health and Nutrition Examination Survey for all citizens. Third, the indicator for socioeconomic levels was subjective thus there may be differences from objective indicators. However subjective economic indicators are also widely used in most studies. Fourth, a survey on the status of persons with disabilities was conducted in 2020, but it was impossible to use this recent data because the 2014 survey only measured the unmet care needs of dental services. It is necessary to suggest including dental indicators in national surveys.

## Conclusions

This study confirmed that the factor most closely related to the unmet dental care needs of disabled people was socioeconomic problems. Its influence also differed by sex and age. Therefore, economic support measures are needed to reduce the unmet dental care needs of disabled people and promote oral health. Long-term policy considerations are required since differences between sexes exist.

### Electronic supplementary material

Below is the link to the electronic supplementary material.

Supplementary Material 1

## Data Availability

The data that supports the findings of this study are available from the Korea Institute for Health and Social Affairs (KIHASA), but restrictions apply to the availability of data, which was used with permission for the current study and therefore not publicly available. The datasets used and analyzed during the current study are available with permission from KIHASA on reasonable request.

## List of Abbreviations

DMFT: Decayed, Missing, Filled Teeth
SES: socioeconomic status
IRB: the institutional review board
KIHASA: Korea Institute for Health and Social Affairs
NHI: National Health Insurance
VIF: Variance inflation factors
SPSS: Statistical Packages for Social Science
KNHANES: Korean National Health and Nutrition Examination Survey
