# Supplementary material for: Is there a sexual difference in the relationship between sociodemographic information and the unmet dental care needs of disabled adults? An analysis from a national survey on persons with disabilities
Source: BMC Oral Health. 2023 Nov 3;23:830. doi: 10.1186/s12903-023-03576-w (PMC10625303; doi:10.1186/s12903-023-03576-w)
Supplement: Supplementary file 1 — Supplementary Material 1 [file 12903_2023_3576_MOESM1_ESM.docx]

Table 1 Act^*^ and enforcement degree of the Act^**^ on welfare of persons with disabilities in South Korea

| ACT ON WELFARE OF PERSONS WITH DISABILITIES  [Enforcement Date 23. Apr, 2013.] [Act No.11521, 22. Oct, 2012., Partial Amendment] | ACT ON WELFARE OF PERSONS WITH DISABILITIES  [Enforcement Date 29. Sep, 2023.] [Act No.19303, 28. Mar, 2023., Partial Amendment] |
| --- | --- |
| Article 2 (Definitions of Persons with Disabilities, etc.) (1) “Person with a disability” means a person whose daily life or social activity is substantially hampered by physical or mental disability over a long period of time.  (2) Among persons with disabilities who fall under paragraph (1), those who are governed by this Act shall have the types and standards of disability prescribed by Presidential Decree and who shall suffer from any of the following conditions:  1. “Physical disability” means a disability of principal external bodily functions and of internal organs, etc.;  2. “Mental disability” means a disability caused by psychological development disorder or mental disease.  (3) “Abuse of persons with disabilities” means physical, mental, emotional or verbal violence, acts of cruelty, economic exploitation, abandonment or negligence towards persons with disabilities. <Newly Inserted by Oct. 22, 2012> | Article 2 (Definitions of Persons with Disabilities, etc.) (1) “Person with a disability” means a person whose daily life or social activity is substantially hampered by physical or mental disability over a long period of time.  (2) Among persons with disabilities who fall under paragraph (1), those who are governed by this Act shall have the types and standards of disability prescribed by Presidential Decree and who shall suffer from any of the following conditions:  1. “Physical disability” means a disability of principal external bodily functions and of internal organs, etc.;  2. “Mental disability” means a disability caused by psychological development disorder or mental disease.  (3) “Abuse of persons with disabilities" means physical, mental, emotional, verbal or sexual violence, acts of cruelty, economic exploitation, abandonment or negligence towards persons with disabilities. <Newly Inserted on Oct. 22, 2012; Jun. 22, 2015>  (4) “Abuse crime of persons with disabilities" means a crime falling under any of the following as abuse of persons with disabilities: <Newly Inserted on Dec. 29, 2020>  1. Among the crimes of homicide under Chapter XXIV, Part II of the Criminal Act, Article 250 (Murder, Killing Ascendant), Article 252 (Murder upon Request or with Consent), Article 253 (Murder upon Request through Fraudulent Means, etc.), and Article 254 (Attempted Crime);  2. Among the crimes of inflicting bodily injury and violence under Chapter XXV of PART II of the Criminal Act, crimes prescribed in Article 257, Article 258, Article 258-2, Article 260 (1) and (2), Article 261 and Article 262;  3. Among the crimes of abandonment and abuse under Chapter XXVIIl of PART II of the Criminal Act, crimes prescribed in Article 271 (1) and (2), Article 272, Article 273 and Article 275;  4. Among the crimes of false arrest and illegal confinement under Chapter XXIX of PART II of the Criminal Act, crimes prescribed in Article 276, Article 277, Article 278, Article 280, and Article 281;  5. Among the crimes of intimidation set forth in Chapter XXX of Part II of the Criminal Act, crimes prescribed in Article 283 (1) and (2), Article 284, and Article 286;  6. Among the crimes of trafficking in persons under Part II, Chapter XXXI of the Criminal Act, crimes prescribed in Article 287, Article 288, Article 289, Article 290, Article 291, Article 292, and Article 294;  7. Among the crimes of rape and indecent conduct set forth in Chapter XXXII of Part II of the Criminal Act, crimes prescribed in Article 297, Article 297-2, Article 298, Article 299, Article 300, Article 301, Article 301-2, Article 302, Article 303, and Article 305;  8. Among the crimes against reputation set forth in Chapter XXXIII of Part II of the Criminal Act, crimes prescribed in Article 307, Article 309, and Article 311;  9. Among the crimes of intrusion upon a human habitation set forth in Chapter XXXVI of Part II of the Criminal Act, crimes prescribed in Article 321;  10. Among the crimes of obstructing another from exercising his or her right set forth in Chapter XXXVII of Part II of the Criminal Act, crimes prescribed in Article 324 and Article 324-5 (limited to crimes falling under Article 324);  11. Among the crimes of fraud and extortion under Chapter XXXIX of Part II of the Criminal Act, crimes prescribed in Article 347, 347-2, 350, 350-2, and 352;  12. Among the crimes of embezzlement and breach of trust under Chapter XL of Part II of the Criminal Act, crimes prescribed in Article 355, Article 356, and Article 357;  13. Among the crimes of destruction and damage set forth in Chapter XLII of Part II of the Criminal Act, crimes prescribed in Article 366;  14. Crimes prescribed in Article 86 (1) and (2), (3) 3 of that Article, (4) 2 and (5) of that Article;  15. Crimes prescribed in Articles 18 and 23 of the Act on the Punishment of Arrangement of Commercial Sex Acts;  16. Crimes prescribed in Article 49 (1) of the Act on the Prohibition of Discrimination against Persons with Disabilities and Remedy against Infringement of Their Rights;  17. Crimes prescribed in Article 70 (1) and (2) of the Act on Promotion of Information and Communications Network Utilization and Information Protection;  18. Crimes prescribed in subparagraphs 1 and 11 of Article 84 of the Act on the Improvement of Mental Health and the Support for Welfare Services for Mental Patients;  19. Crimes provided for in subparagraphs 1 through 18, which are subject to aggravated punishment under other Acts. |
| Article 32 (Registration as Persons with Disabilities) (1) A person with a disability and his or her legal representative or his or her guardian prescribed by Presidential Decree (hereinafter referred to as "legal representative, etc.") shall register details of the disability and other matters prescribed by Ordinance of the Ministry of Health and Welfare with the Special Self-Governing City Mayor, the Special Self-Governing Province Governor, or the head of a Si/Gun/Gu (the head of a Gu refers to the head of an autonomous Gu; hereinafter the same shall apply), and if the person with a disability who has filed an application for registration meets the standards referred to in Article 2, the Special Self-Governing City Mayor, the Special Self-Governing Province Governor, or the head of the Si/Gun/Gu shall issue a registration certificate for a person with a disability (hereinafter referred to as "registration certificate") to him or her. <Amended on Feb. 29, 2008; Jan. 18, 2010; May 27, 2010; Jun. 22, 2015; Feb. 8, 2017>  (2) Deleted. <Feb. 8, 2017>  (3) The Special Self-Governing City Mayor, the Special Self-Governing Province Governor, and the head of a Si/Gun/Gu may take necessary measures for a person with a disability or his or her legal representative, etc., such as diagnosis of disability, in order to adjust the degree of disability of the person with a disability who has received a registration certificate pursuant to paragraph (1) according to a change in the conditions of disability. <Amended on Feb. 8, 2017; Dec. 19, 2017>  (4) The Ministry of Health and Welfare may have a Disability Decision Committee under its jurisdiction to take charge of the affairs concerning disability acknowledgement and assessment. <Amended on Feb. 29, 2008; Jan. 18, 2010; Dec. 19, 2017>  (5) No registration certificate shall be transferred or lent, and no name or mark similar to a registration certificate shall be used.  (6) The Special Self-Governing City Mayor, the Special Self-Governing Province Governor, and the head of a Si/Gun/Gu may request a precise examination on the degree of disability from a public institution under Article 4 of the Act on the Management of Public Institutions, as prescribed by Presidential Decree, if necessary to verify that the disability acknowledgement and assessment of a person with a disability is appropriate in registering the person with a disability under paragraph (1) and in adjusting the degree of disability according to a change in the conditions of disability under paragraph (3). <Newly Inserted on May 27, 2010; Jun. 22, 2015; Dec. 29, 2015; Dec. 19, 2017>  (7) A public institution entrusted with a precise examination on the degree of disability under paragraph (6) may request the perusal of materials related to the relevant medical services of the person in question and the issuance of the copy thereof from a medical institution under the Medical Service Act after obtaining consent from the person who intends to receive the examination, his/her legal representative, etc., if necessary. In such cases, the medical institution in receipt of such request shall comply therewith, unless there is a good reason for not doing so, and the State or a local government may assist with fees, charges, etc. for the use of materials provided to such public institution within the budget. <Newly Inserted by Dec. 29, 2015; Feb. 8, 2017>  (8) Except as provided in paragraphs (1) and (3) through (7), matters necessary for registration of persons with disabilities, the issuance of registration certificates, the diagnosis of disabilities and precise examinations on the degree of disability, the Disability Decision Committee, the request for the perusal of materials related to medical services or for the issuance of the copies thereof, and other relevant matters shall be prescribed by Ordinance of the Ministry of Health and Welfare. <Amended by Feb. 29, 2008; Jan. 18, 2010; May 27, 2010; Dec. 29, 2015; Feb. 8, 2017> | Article 32 (Registration as Persons with Disabilities) (1) A person with a disability and his or her legal representative or his or her guardian prescribed by Presidential Decree (hereinafter referred to as "legal representative, etc.") shall register details of the disability and other matters prescribed by Ordinance of the Ministry of Health and Welfare with the Special Self-Governing City Mayor, the Special Self-Governing Province Governor, or the head of a Si/Gun/Gu (the head of a Gu refers to the head of an autonomous Gu; hereinafter the same shall apply), and if the person with a disability who has filed an application for registration meets the standards referred to in Article 2, the Special Self-Governing City Mayor, the Special Self-Governing Province Governor, or the head of the Si/Gun/Gu shall issue a registration certificate for a person with a disability (hereinafter referred to as "registration certificate") to him or her. <Amended on Feb. 29, 2008; Jan. 18, 2010; May 27, 2010; Jun. 22, 2015; Feb. 8, 2017>  (2) Deleted. <Feb. 8, 2017>  (3) The Special Self-Governing City Mayor, the Special Self-Governing Province Governor, and the head of a Si/Gun/Gu may take necessary measures for a person with a disability or his or her legal representative, etc., such as diagnosis of disability, in order to adjust the degree of disability of the person with a disability who has received a registration certificate pursuant to paragraph (1) according to a change in the conditions of disability. <Amended on Feb. 8, 2017; Dec. 19, 2017>  (4) The Ministry of Health and Welfare may have a Disability Decision Committee under its jurisdiction to take charge of the affairs concerning disability acknowledgement and assessment. <Amended on Feb. 29, 2008; Jan. 18, 2010; Dec. 19, 2017>  (5) No registration certificate shall be transferred or lent, and no name or mark similar to a registration certificate shall be used.  (6) The Special Self-Governing City Mayor, the Special Self-Governing Province Governor, and the head of a Si/Gun/Gu may request a precise examination on the degree of disability from a public institution under Article 4 of the Act on the Management of Public Institutions, as prescribed by Presidential Decree, if necessary to verify that the disability acknowledgement and assessment of a person with a disability is appropriate in registering the person with a disability under paragraph (1) and in adjusting the degree of disability according to a change in the conditions of disability under paragraph (3). <Newly Inserted on May 27, 2010; Jun. 22, 2015; Dec. 29, 2015; Dec. 19, 2017>  (7) Deleted. <Jul. 27, 2021>  (8) Except as provided in paragraphs (1) and (3) through (6), matters necessary for registration of persons with disabilities, the issuance of registration certificates, the diagnosis of disabilities and precise examinations on the degree of disability, the Disability Decision Committee, and other relevant matters shall be prescribed by Ordinance of the Ministry of Health and Welfare. <Amended on Feb. 29, 2008; Jan. 18, 2010; May 27, 2010; Dec. 29, 2015; Feb. 8, 2017; Jul. 27, 2021> |

| ENFORCEMENT DECREE OF THE ACT ON WELFARE OF PERSONS WITH DISABILITIES  [Enforcement Date 01. Jan, 2019.] [Presidential Decree No.29421, 24. Dec, 2018., Amendment by Other Act] | ENFORCEMENT DECREE OF THE ACT ON WELFARE OF PERSONS WITH DISABILITIES  [Enforcement Date 28. Jan, 2022.] [Presidential Decree No.32364, 25. Jan, 2022., Partial Amendment] |
| --- | --- |
| Article 2 (Types and Standards of Disabilities) (1) "Persons falling into the types and standards prescribed by Presidential Decree" in Article 2 (2) of the Act on Welfare of Persons with Disabilities (hereinafter referred to as the "Act") means the persons prescribed in attached Table 1.  (2) Persons with disabilities shall be rated by degree of disability, on condition that such ratings shall be prescribed by Ordinance of the Ministry of Health and Welfare. <Amended by Feb. 29, 2008; Mar. 15, 2010> | Article 2 (Types and Standards of Disabilities) (1) "Persons falling into the types and standards prescribed by Presidential Decree" in the provisions, with the exception of the subparagraphs, of Article 2 (2) of the Act on Welfare of Persons with Disabilities (hereinafter referred to as the "Act") means the persons prescribed in attached Table 1. <Amended on Dec. 31, 2018>  (2) The degree of disabilities shall be prescribed by Ordinance of the Ministry of Health and Welfare. <Amended on Dec. 31, 2018>  [Title Amended on Dec. 31, 2018] |
| Table 1. Disability grade for persons with disabilities (related to Article 2) [a part of whole]  1. People with a physical disability  A. A person who has lost a part of his body.  Grade 1  1. A person who has lost both arms in areas above the wrist joint.  2. A person who has lost both legs in areas above the knee joint.  Grade 2  1. A person who lost both fingers of his hands  2. A person who has lost one arm above the elbow joint.  3. A person who has lost both legs in areas above the ankle joint.  Grade 3  1. A person who lost the thumb and second finger of both hands  2. A person who has lost every finger in one hand.  3. A person who has lost both legs in areas above the Chopart's joint.  4. A person who has lost one leg above the knee joint.  Grade 4  1. A person who lost the thumb of both hands  2. a person who has lost a thumb and a second finger.  3. A person who has lost three fingers, including a thumb on one hand.  4. A person who has lost both legs in areas above the Lisfranc joint (joint connecting the dorsal bones and ankles)  5. A person who has lost one leg above the ankle joint.  Grade 5  1. A person who has lost two fingers, including one thumb.  2. A person who has lost the thumb of one hand in an area above the mesenchymal joint.  3. A person who has lost three fingers, including the second finger of one hand.  4. A person who lost both toes  5. A person who has lost one leg in an area above the sofa joint.  Grade 6  1. Put your thumb on one hand a lost man.  2. A person who has lost two fingers, including the second finger of one hand.  3. A person who has lost all of the third, fourth, and fifth fingers of one hand.  4. A person who has lost one leg above the Lysfranc joint. | Table 1. Disability severity for persons with disabilities (related to Article 2)  1. People with a physical disability  A. A person who has lost a part of his body.  1) A person with a severe disability  A) A person who lost the thumb and second finger of both hands  B) A person who has lost all fingers in one hand  C) A person who has lost both legs in areas above the Chopart's joint  D) A person who has lost one leg in an area above the knee joint  2) a person with not a severe of disability  A) a person who lost a thumb on one hand  B) A person who has lost two fingers, including the second finger of one hand  C) A person who has lost all of the third, fourth, and fifth fingers of one hand  D) A person who has lost one leg above the lisprank joint of the ankle  E) a person who has lost both toes |

* This Act explains the definitions and registration of Persons with Disabilities in South Korea.

** This enforcement degree of the Act explains the types and standards of persons with disabilities. As shown on the left side of the table, the grading system was applied at the time of the 2014 national survey on persons with disabilities. However, this grading system changed into a severity system in 2022.
